# The VIP/VPAC1R Pathway Regulates Energy and Glucose Homeostasis by Modulating GLP-1, Glucagon, Leptin and PYY Levels in Mice

**DOI:** 10.3390/biology11030431

**Published:** 2022-03-11

**Authors:** Daniel Sanford, Leon Luong, John P. Vu, Suwan Oh, Arielle Gabalski, Michael Lewis, Joseph R. Pisegna, Patrizia Germano

**Affiliations:** 1 Research Service, Veterans Affairs Greater Los Angeles Healthcare System, Los Angeles, CA 90073, USA; danielsanford1@yahoo.com (D.S.); leonluong3@gmail.com (L.L.); john.p.vu@gmail.com (J.P.V.); suwanoh@gmail.com (S.O.); gabalski.a@gmail.com (A.G.); 2 Digestive Diseases Research Center (CURE), Department of Medicine, University of California, Los Angeles, CA 90073, USA; joseph.pisegna@va.gov; 3 Department of Pathology, VA Greater Los Angeles Healthcare System, Los Angeles, CA 90073, USA; michael.lewis8@va.gov; 4 Division of Gastroenterology, Hepatology and Parenteral Nutrition, Department of Medicine, VA Greater Los Angeles Healthcare System, Los Angeles, CA 90073, USA; 5 Department of Human Genetics, David Geffen School of Medicine at UCLA, Los Angeles, CA 90095, USA; 6 Division of Pulmonary and Critical Care, Veterans Affairs Greater Los Angeles Healthcare System, Los Angeles, CA 90073, USA

**Keywords:** VPAC1R, VIP, calorimetry, glucose, GLP-1, glucagon, leptin, PYY

## Abstract

**Simple Summary:**

The current study is the first complete characterization of the phenotypic, metabolic, calorimetric, and homeostatic effects of VPAC1R in a null murine model. To evaluate the role of VPAC1R on body phenotype, feeding behavior, glucose/energy homeostasis, metabolic rate and plasma hormones, a long-term study was conducted in VPAC1R^−/−^ and WT mice. The outcome data document that VPAC1R^−/−^ mice have altered metabolism and insulin intolerance, with significant increase of feeding bouts, reduction of total energy expenditure and respiratory gases during both the dark and light cycle, together with elevated fasting levels of GLP-1 and PYY, and higher postprandial levels of GLP-1, glucagon leptin and PYY. These findings suggests that VPAC1R controls glucose homeostasis and energy balance by regulating plasma metabolic hormones.

**Abstract:**

Vasoactive Intestinal Peptide binds with high affinity to VPAC1R and VPAC2R, thus regulating key physiologic functions. Previously, we documented in VIP^−/−^ mice a leaner body phenotype and altered metabolic hormones. Past reports described in VPAC2^−/−^ mice impaired circadian rhythm, reduced food intake, and altered metabolism. To better define the effects of VPAC1R on body phenotype, energy/glucose homeostasis, and metabolism, we conducted a 12-week study in a VPAC1R null model. Our results reveal that VPAC1^−/−^ mice experienced significant metabolic alterations during the dark cycle with greater numbers of feeding bouts (*p* = 0.009), lower Total Energy Expenditure (*p* = 0.025), VO_2_ (*p* = 0.029), and VCO_2_ (*p* = 0.016); as well as during the light cycle with lower Total Energy Expenditure (*p* = 0.04), VO_2_ (*p* = 0.044), and VCO_2_ (*p* = 0.029). Furthermore, VPAC1^−/−^ mice had significantly higher levels of GLP-1 and PYY during fasting, and higher levels of GLP-1, glucagon leptin and PYY during postprandial conditions. In addition, VPAC1^−/−^ mice had lower levels of glucose at 60′ and 120′, as assessed by insulin tolerance test. In conclusion, this study supports a key role for VPAC1R in the regulation of body glucose/energy homeostasis and metabolism.

## 1. Introduction

Gastrointestinal neuropeptides are known to regulate important physiological functions such as appetite/satiety, feeding behavior, digestion, nutrient absorption, energy expenditure, water balance, glucose homeostasis, and immunomodulation [1,2,3,4,5]. These neuropeptides, localized both centrally and peripherally, bind to receptors distributed throughout the central nervous system, peripheral nerves and tissues, as in the gastrointestinal (GI) tract. Among these neuropeptides, Vasoactive Intestinal Peptide (VIP) has been widely studied and found to be implicated in the regulation of gastrointestinal physiology and homeostatic functions including vasodilation, microbiota, sphincter relaxation, mucosal ion transport, motility and secretion, appetite, feeding behavior, inflammatory reactions, and immunity [6,7,8,9,10,11,12,13,14,15].

We have previously demonstrated the regulatory functions of endogenous VIP on appetite, body phenotype, and metabolism in VIP^−/−^ mice [16]. Our long-term study showed that VIP^−/−^ mice, compared to their WT littermates, had leaner body phenotypes with a deficit of fat mass accumulation as they aged, and a better maintained body lean mass along with altered plasma levels of adiponectin, GLP-1, leptin, PYY and insulin [16]. Overall, our study showed that endogenous VIP is a key regulator of appetite, feeding behavior, body phenotype, and plasma metabolic hormone levels, thus supporting a potential role for the VIP pathways in the development of obesity and metabolic syndrome [16]. However, the major limitation of this study was that inability to evaluate VPAC1R mediated effects due to the potential binding of PACAP, a closely related peptide which has affinity for VPAC1R. To overcome this issue, here, we utilized a VPAC1R null model.

VIP binds to two different class B G-protein coupled receptors (GPCRs), VPAC1 and VPAC2 [17,18], each structurally defined by having seven transmembrane helices and signaling via a Gas/cAMP pathway [19,20]. The “classical” VIP receptor, VPAC1R, is widely distributed throughout the GI tract and associated organs, including stomach, liver, and pancreas [21,22,23,24,25]. In the small intestine and colon lumen/endothelium, VPAC1R has mRNA expression levels which are almost 300 times higher than VPAC2R [21,22,23,26]. In the human small intestine and colonic epithelium, VPAC1R was demonstrated to have a 4-fold greater expression than VPAC2R [22]. Furthermore, VPAC1R were shown to be involved in intestinal secretion and longitudinal muscle contraction in the jejunum [27]. The recent development of a genetically engineered VPAC1^−/−^ mouse model has allowed for better analysis of the pathways and physiological processes which are mediated by VPAC1R [28].

In order to achieve our aim to clarify the role of VPAC1R in the regulation of appetite/satiety, feeding behavior, body phenotype, metabolism and energy homeostasis, we have conducted a long-term metabolic study of VPAC1^−/−^ mice using an Echo-MRI body analyzer, a Promethion metabolic system, and glucose and metabolic hormone assays. Our results show that genetic lack of VPAC1R induced significant alterations in respiratory gases, total energy expenditure (TEE), plasma metabolic hormone levels, and glucose homeostasis, but it did not significantly affect body weight and mass composition, food intake, physical activity and the metabolic RQ index in mice. These findings support a key role for VPAC1R in the regulation of body glucose, energy homeostasis and plasma metabolic hormone levels.

## 2. Materials and Methods

### 2.1. Animals and Diets

VPAC1R null (VPAC1R^−/−^) mice, backcrossed > 12 generations to C57BL/6J mice, were obtained from Dr. D’Odorisio [28]. Age-matched wild-type (WT) littermates from the same colony were group-housed and fed ad libitum in a specific pathogen-free, sterile animal facility, under conditions of controlled illumination (12:12-h light–dark cycle; lights from 06:00 to 18:00 h) at the United States Department of Veteran Affairs Greater Los Angeles Healthcare System animal facility. The animal protocol of this experimental study (#06010-17) was approved by the Institutional Animal Care and Use Committee (IACUC) of the VA Greater Los Angeles Healthcare System.

### 2.2. Experimental Design

VPAC1^−/−^ male mice (8 weeks of age) and their age- and sex-matched WT littermates were divided into two experimental groups (*n* = 8/group). Body weight and mass composition were monitored for 12 weeks. After this period, mice were transferred and single-housed in Promethion metabolic cages, allowed to acclimate for a 5-day period, and then their indirect calorimetric parameters were recorded and analyzed. Plasma samples were obtained from each experimental mouse in fasting and post-prandial conditions to assess the levels of a panel of metabolic hormones. Glucose tolerance test and insulin tolerance test were conducted in each studied mouse, as previously published [16,29].

### 2.3. Body Phenotype Analysis

VPAC1^−/−^ (*n* = 8) and WT mice (*n* = 8) were group-housed (4 animals per cage) and given free access to water and standard rodent diet (Prolab RMH 2500; LabDiet, St. Louis, MO, USA). Murine body weight and mass composition were measured at baseline and then bi-weekly by using an Echo-MRI 4 in 1 whole body composition analyzer (EchoMedical Systems, Houston, TX, USA). Body weight, fat, and lean mass changes were calculated in terms of percentage of change from the baseline measurement at the beginning of the study.

### 2.4. Determination of Food Intake Behavior

Analyses of food and water intake were performed using an automated Promethion Metabolic system (Sable Systems, Las Vegas, NV, USA), allowing for continuous, undisturbed, real-time monitoring of the food hopper and water bottle [30,31,32], as previously published [16,29]. Mice were single-housed, and after a five-day acclimation period, complete food/water consumption, feeding and drinking behavior were characterized for a 24-h period. Cumulative food/water intake, bout/meal frequency, total time spent eating and drinking (time in minutes or percentages), bout/meal size, duration, and eating rate were measured. “Mean food intake” is defined as the total food intake measured during a period divided by the number of bouts and the number of minutes that each animal spent eating. Food intake was measured undisturbed in real time through a weight sensor with a 3 mg resolution, which was attached to a food hopper. Total water intake was calculated as total grams of water consumed during either the dark or light phase. Bouts are defined as the number of times that each mouse drank water or consumed food. Mean water intake was calculated as total water intake measured during a period divided by the number of bouts and minutes that each animal spent drinking.

### 2.5. Assessment of Indirect Calorimetry Using the Promethion Metabolic System

Indirect calorimetry data were recorded in each experimental animal by using a Promethion Metabolic Cage System (Sable Systems, Las Vegas, NV, USA) [5,16,29]. Mice were single-housed and acclimated for five days in Promethion metabolic cages. Then, respiratory gases and water vapor were measured with an integrated fuel cell oxygen analyzer, spectrophotometric CO_2_ analyzer, and a capacitive water vapor partial pressure analyzer [30]. Respiratory quotient (RQ) was calculated as the ratio of CO_2_ production over O_2_ consumption. Energy expenditure was calculated using the Weir equation: kcal/h = 60 × (0.003941 × VO_2_ + 0.001106 × VCO_2_) [31]. These parameters were measured over a 48-h period in all experimental animals.

### 2.6. Plasma Samples and Measurement of Metabolic Hormone Levels 

Following the EchoMRI body composition analysis and Promethion metabolic cage study, blood samples were obtained from VPAC1^−/−^ (*n* = 8) and WT (*n* = 8) mice by retroorbital collection using a 75 uL heparinized tube (Fisherbrand, Fisher Scientific) and an EDTA microcollection tube (Microtainer, Becton Dickenson) from live mice, anesthetized with inhaled isoflurane after overnight fasting (18:00–06:00 h) and in postprandial conditions, consisting of overnight fasting (18:00–06:00 h) followed by re-feeding period (06:00–10:00 h), with blood being collected 1 h after feeding.

Plasma samples, obtained from the whole blood collection, were added with a cocktail of protease inhibitors, including inhibitor cocktail tablets with EDTA (Roche, Indianapolis, IN), aprotinin (Pittsburgh, PA, USA), and dipeptidyl peptidase IV (DPP-IV) inhibitor (Millipore, Billerica, MA, USA), and stored frozen at −80 °C until the assay was performed. Plasma levels of a panel of metabolic hormones including active ghrelin, GLP-1, glucagon, insulin, leptin, and peptide YY (PYY) were assessed, using a Milliplex MAP Mouse Metabolic Hormone Magnetic Bead Panel (Millipore, Billerica, MA, USA) according to the manufacturer’s instructions, as previously published [5,16,29]. Analysis of quality control standards provided in the kit matched expectations, and the assay had an inter-assay precision of <25% and an intra-assay precision of <7%.

### 2.7. Insulin Tolerance and Glucose Tolerance Test

Glucose tolerance test: mice were fasted overnight (18:00–09:00 h), then dextrose (Sigma) was injected intraperitoneally (2 U/kg), and glucose levels in the tail vein blood were measured at 0, 15, 30, 60, and 120 min by using a OneTouch ultra-meter, as previously published [5,16,29].

Insulin tolerance test: mice were fasted overnight (18:00–09:00 h), then insulin (Sigma) was injected intraperitoneally (0.75 U/kg), and glucose levels in the tail vein blood were measured at 0, 15, 30, 60, and 120 min by using a OneTouch ultra-meter, as previously published [5,16,29]. Fasting blood glucose measurements were also performed using the same system.

### 2.8. Data Analysis and Trial Exclusion

A two-way analysis of variance (ANOVA) with a Sidak’s multiple comparisons post hoc test was used to evaluate the statistical significance of RQ, VO_2_, VCO_2_, and TEE between the two experimental treatment groups. Factors included in the two-way ANOVA were: genetic strain (VPAC1^−/−^, WT), categorical time (X-Minute Intervals), and an interaction term (food intake by time). The area under the curve (AUC) for RQ, VO_2_, VCO_2_, and TEE values were calculated, and a *t*-test was used to determine the statistical significance between the two treatment groups. Food and water consumption were analyzed by multiple *t*-test. Metabolic hormone levels were analyzed using a two-way ANOVA with a Sidak’s multiple comparisons post hoc test. Glucose tolerance and insulin tolerance curve values were statistically analyzed using an unpaired *t*-test. Graphs were constructed and standard error of the mean values plotted using GraphPad Prism 6/7 Software (La Jolla, CA, USA).

## 3. Results

### 3.1. Body Phenotype Long-Term Study in VPAC1^−/−^ Mice

**Figure 1:**
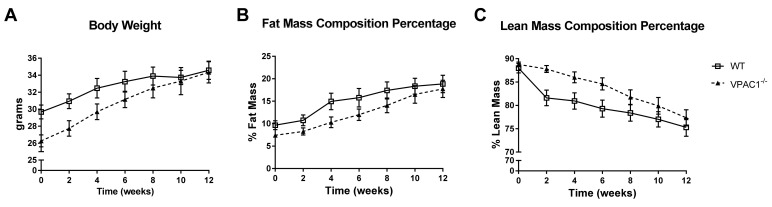
A 12-week analysis of body weight (g) (**A**), fat mass % (**B**), and lean mass % (**C**) was conducted in VPAC1^−/−^ (closed triangle) and WT mice (open square). Unpaired *t*-test was used for the data analysis; Values are means ± SEMs.

In VPAC1^−/−^ mice (*n* = 8) and WT mice (*n* = 8), fed a standard diet *ad libitum* for 12 weeks, an analysis of body phenotype was conducted weekly throughout the study period. The results showed no significant differences between the two groups, even though at the beginning of the study, VPAC1^−/−^ mice had lower, but not significant, body weight values compared to WT littermates fed with the same diet (26.3 ± 1.5 vs. 29.7 ± 1.4 g) (Figure 1A). Body fat and lean mass analysis, measured as percentage of change from the first measurement obtained at 8 weeks of age, confirmed that VPAC1^−/−^ mice had no significant differences in body fat mass (Figure 1B) and lean mass compared to WT mice (Figure 1C). The values were measured by Echo-MRI at two-week intervals throughout the study period.

### 3.2. Feeding Behavior and Food Intake in VPAC1^−/−^ Mice

**Figure 2:**
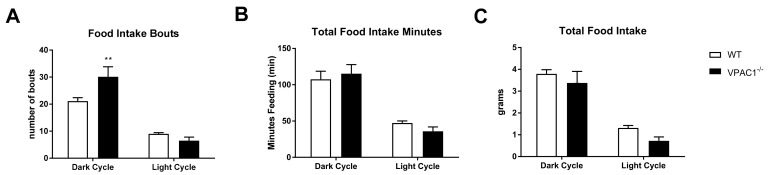
A 24-h analysis of food intake bouts (**A**), total food intake minutes (**B**), and total food intake grams (**C**) was conducted in VPAC1^−/−^ and WT mice. Values were separated into dark (active) cycle and light (dormant) cycle. A two-factor ANOVA analysis was used to compare number of bouts, duration of bouts, and amount of food consumed. Values are means ± SEMs; ** *p* < 0.01.

At the end of the 12-week study period, feeding behavior and food intake were assessed in each VPAC1^−/−^ and WT experimental animal using a Promethion metabolic cage system. Food consumption data of VPAC1^−/−^ and WT mice were calculated in terms of 24 h average values over 48 h of uninterrupted recording. The detailed time course of the cumulative food intake is illustrated in Figure 2. The recorded data show that VPAC1^−/−^ mice had a significantly greater number of feeding bouts during the active phase (dark cycle) as compared to their WT littermates (30.13 ± 3.69 bouts vs. 21.13 ± 1.27 bouts, *p* = 0.009); however, no significant differences were found during the dormant phase (light cycle) between the two experimental groups (Figure 2A). Furthermore, no significant differences were recorded between the two groups in the total number of minutes spent eating (*p* = 0.8) (Figure 2B), as well as in the total grams of food consumed (*p* = 0.5) (Figure 2C), during either the dark or the light phase of the cycle.

### 3.3. Calorimetric Parameters Alteration in VPAC1^−/−^ Mice

**Figure 3:**
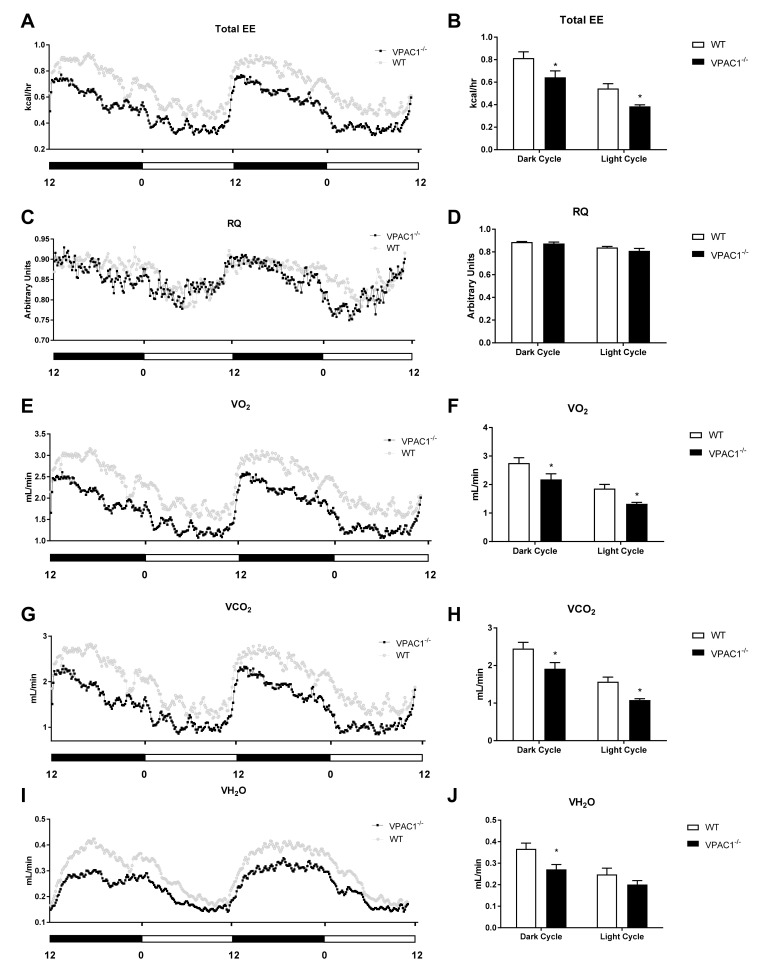
Analyses of respirometry and calorimetry data in VPAC1^−/−^ and WT mice: TEE (**A**) and dark phase and light phase averages of TEE (**B**); RQ (**C**) and dark phase and light phase averages of RQ (**D**); VO_2_ (**E**) and dark phase and light phase averages of VO_2_ (**F**); VCO_2_ (**G**) and dark phase and light phase averages of VCO_2_ (**H**); VH_2_O (**I**) and dark phase and light phase averages of VH_2_O (**J**). A two-factor ANOVA analysis was used to compare TEE, RQ, VO_2_, VCO_2_, and VH_2_O dark phase and light phase averages. In panels **B**, **D**, **F**, **H**, and **J**, values are means ± SEMs; * *p* < 0.05.

Indirect calorimetric analyses were conducted over a 24-h period (Figure 3) in VPAC1^−/−^ mouse (*n* = 8) and WT littermates (*n* = 8), single-housed in Promethion metabolic cages; the data revealed a difference in Total Energy Expenditure (TEE), as assessed by analyzing two full light/dark cycles (Figure 3A). VPAC1^−/−^ mice had significantly lower TEE values than WT littermates, as assessed using an area under the curve (AUC) analysis, during both the dark phase (0.643 ± 0.058 kcal/hr vs. 0.814 ± 0.055 kcal/hr, *p* = 0.025) and the light phase of the cycle (0.385 ± 0.014 kcal/hr vs. 0.544 ± 0.042, *p* = 0.0402) (Figure 3B).

Respiratory Quotient (RQ) values, calculated in both VPAC1^−/−^ and WT mice as a ratio between released CO_2_ (VO_2_) and consumed O_2_ (VO_2_), showed no significant differences between VPAC1^−/−^ and WT experimental mice (Figure 3C), as confirmed by AUC analysis (Figure 3D). Respiratory gases (VO_2_ and VCO_2_) were continuously measured in each experimental VPAC1^−/−^ and WT mouse during two full light and dark cycles, as shown, respectively, in Figure 3E,G. VPAC1^−/−^ mice had significantly lower VO_2_ values, compared to WT mice, during the dark phase (2.181 ± 0.197 mL/min vs. 2.756 ± 0.187 mL/min, *p* = 0.029) as well as the light phase (1.325 ± 0.051 mL/min vs. 1.86 ± 0.145 mL/min, *p* = 0.044) (Figure 3F). Furthermore, VPAC1^−/−^ mice had significantly lower VCO_2_ values than WT littermates during the dark phase (1.914 ± 0.164 mL/min vs. 2.451 ± 0.165 mL/min, *p* = 0.016) as well as the light phase (1.081 ± 0.036 mL/min vs. 1.57 ± 0.122 mL/min, *p* = 0.029) (Figure 3H). The water vapor (VH_2_O) released by VPAC1^−/−^ and WT mice was also measured over 48 h two full light/dark cycles (Figure 3I). In VPAC1^−/−^ mice, significantly lower levels of released VH_2_O were detected, compared to WT mice, during the dark phase (0.271 ± 0.022 mL/min vs. 0.367 ± 0.027 mL/min, *p* = 0.0198); however, no significant differences were found during the light phase (*p* = 0.33) (Figure 3J).

### 3.4. Physical Activity Values in VPAC1^−/−^ Mice

**Figure 4:**
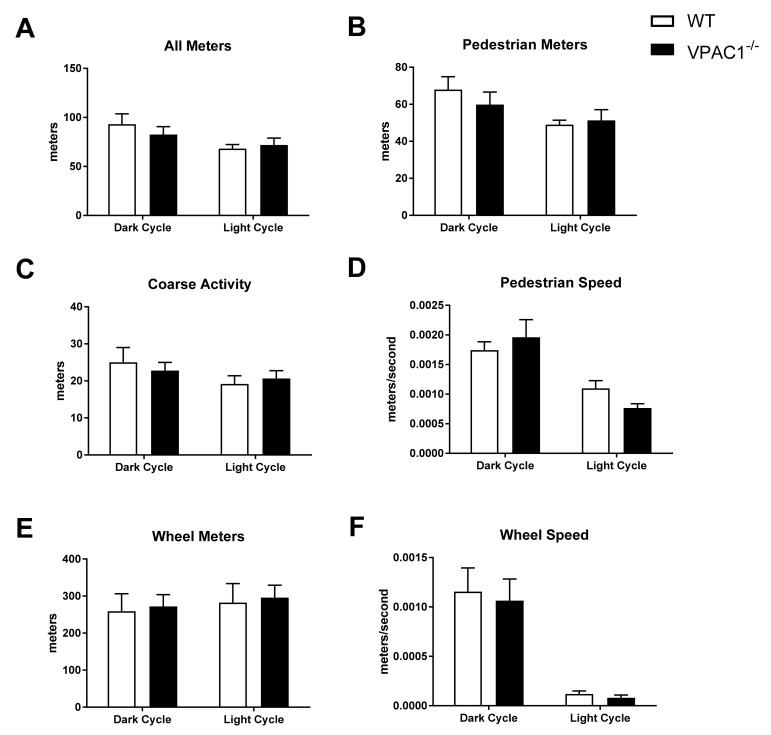
Analysis of physical activity and movement in WT mice and VPAC1^−/−^ mice: All meters (total activity) dark phase and light phase averages (**A**); Pedestrian meters (mouse walking) dark phase and light phase averages (**B**); Course activity (mouse movement) dark phase and light phase averages (**C**); Pedestrian speed (mouse speed) dark phase and light phase averages (**D**); Wheel meters (rotation) dark phase and light phase averages (**E**); Wheel speed (rotation speed) dark phase and light phase averages (**F**). A two-factor ANOVA analysis was used to compare these dark phase and light phase averages. Values in panels A through F are means ± SEMs.

In VPAC1^−/−^ (*n* = 8) and WT mouse (*n* = 8), single-housed in Promethion metabolic cages, physical activity profiles were analyzed over a 24-h dark/light cycle period (Figure 4). All meters (total activity) in Figure 4A, pedestrian meters (mouse walking) in Figure 4B, coarse activity (mouse movement) in Figure 4C, pedestrian speed (mouse speed) in Figure 4D, wheel meters (rotation) in Figure 4E, and wheel speed (rotation speed) in Figure 4F, were assessed. No significant differences were found between VPAC1^−/−^ and WT mice in any of the aforementioned parameters during the dark phase as well as during the light phase.

### 3.5. Circulating Levels of Metabolic Hormones Are Dysregulated in VPAC1^−/−^ Mice

**Figure 5:**
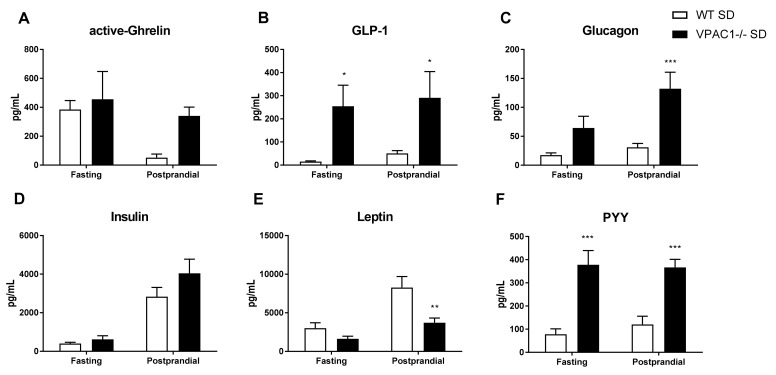
Plasma levels of a panel of orexigenic and anorexigenic metabolic hormones assayed in WT mice and VPAC1^−/−^ mice: active-Ghrelin (**A**), GLP-1 (**B**), glucagon (**C**), insulin (**D**), leptin (**E**), and PYY (**F**) were measured in either fasting or postprandial conditions. A 2-factor ANOVA analysis was used to compare averages. Values are means ± SEMs. * *p* < 0.05, ** *p* < 0.01, *** *p* < 0.001.

To better elucidate the effects of the VPAC1R on metabolism, the plasma levels of a panel of orexigenic-anorexigenic metabolic hormones were measured in VPAC1^−/−^ and WT mice (Figure 5) during fasting, as well as in postprandial conditions, as shown in Figure 5A. No significant differences in active-ghrelin levels were detected between the two experimental groups, although under postprandial conditions, the higher ghrelin values measured in VPAC1^−/−^ mice were approaching significance (*p* = 0.08). In VPAC1^−/−^ mice, circulating levels of GLP-1 were significantly higher during fasting (254.30 ± 91.43 pg/mL vs. 15.41 ± 2.81 pg/mL, *p* = 0.035) as well as during postprandial conditions (291.19 ± 113.24 pg/mL vs. 50.62 ± 11.93 pg/mL, *p* = 0.0287), as shown in Figure 5B. Plasma levels of glucagon demonstrated no significant differences (*p* = 0.12) between VPAC1^−/−^ and WT mice (Figure 5C) during fasting conditions; however, significantly higher postprandial glucagon values were measured in VPAC1^−/−^ mice (132.28 ± 28.43 pg/mL vs. 30.99 ± 6.56 pg/mL, *p* = 0.0006). No significant differences in plasma levels of insulin were found under fasting or postprandial conditions between the two experimental groups (Figure 5D). Plasma leptin showed no significant differences during fasting conditions; however, significantly lower postprandial leptin levels were found in VPAC1^−/−^ mice (3717.125 ± 599.77 pg/mL vs. 8272.57 ± 1415.25 pg/mL, *p* = 0.0011), as shown in Figure 5E. Circulating levels of PYY (Figure 5F) were significantly higher in VPAC1^−/−^ mice during fasting (378.00 ± 174.03 pg/mL vs. 78.32 ± 69.13 pg/mL, *p* < 0.0001), as well as in postprandial conditions (366.88 ± 97.33 pg/mL vs. 120.76 ± 105.66 pg/mL, *p* = 0.0012).

### 3.6. VPAC1^−/−^ Mice Have Altered Insulin Sensitivity and Tolerance

**Figure 6:**
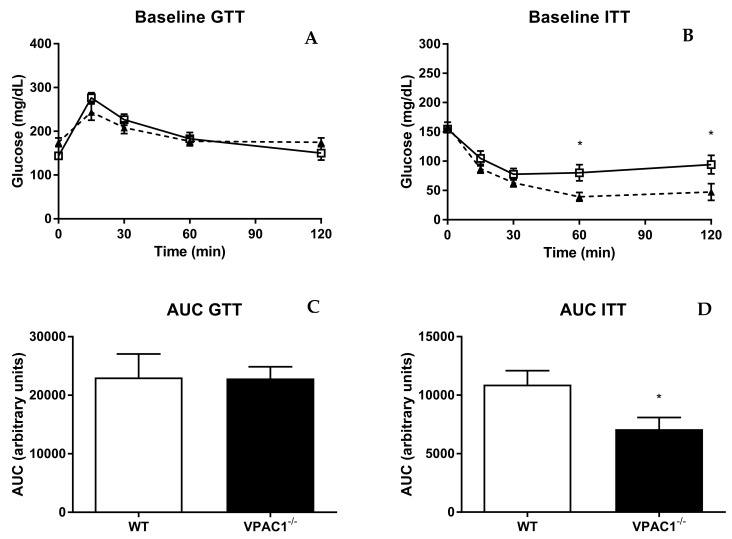
Glucose tolerance test (GTT) and insulin tolerance test (ITT) in WT (open square) and VPAC1^−/−^ mice (closed triangle). Blood glucose values (**A**,**C**) and area under the curve (AUC) of glycemia analysis (**B**,**D**) of blood glucose levels. An unpaired *t*-test was used to compare time points post glucose/insulin infusion in WT mice and VPAC1^−/−^ mice. A two-factor ANOVA analysis was used to compare AUC values between WT and VPAC1^−/−^ mice. Values are means ± SEMs; * *p* < 0.05.

Glucose tolerance and insulin sensitivity were studied in VPAC1^−/−^ and WT mice (Figure 6). The glucose tolerance test (GTT) revealed no significant differences between VPAC1^−/−^ and WT mice at 0, 30, 60, and 120 min, post glucose infusion (Figure 6A), as shown also by the area under the curve values (AUC) (Figure 6B). Insulin tolerance test (ITT), conducted in VPAC1^−/−^ and WT mice, demonstrated significantly lower glucose levels in VPAC1^−/−^ mice, compared to WT mice, at 60 min (80.14 ± 13.65 mg/dL vs. 39.00 ± 7.62 mg/dL, *p* = 0.0419) and at 120 min (94.00 ± 15.89 mg/dL vs. 47.29 ± 14.46 mg/dL, *p* = 0.0152), as seen in Figure 6C. No significant differences in glucose levels were measured at 0, 15, and 30 min, post insulin infusion (Figure 6C). The area under the curve analysis (Figure 6D) confirms a significant decrease in plasma glucose levels in VPAC1^−/−^ mice as compared to WT mice (10903 ± 1185 AU vs. 7094 ± 995 AU, *p* = 0.0299).

## 4. Discussion

VIP is most highly expressed in the gut, in which it regulates motility, digestive processes, absorption, secretion, microbiota, and immunity [6,7,8,9,10,11,12,13,14,15]. Previously, we assessed the overall physiologic functions of endogenous VIP on body phenotype and metabolism [16] and suggested that alteration of the VIP pathways could be involved in the development of obesity and metabolic syndrome. VIP, as well as PACAP neuropeptide, binds with similar high affinity to VPAC1 and VPAC2 receptors [17,18,19,20]. Its role on glucose/energy homeostasis and metabolism has not been fully established, therefore, the main aim of our current research is to establish VPAC1R effects on body and energy homeostasis and metabolism.

In this study, a 12-week phenotypic analysis of age-and sex-matched VPAC1^−/−^ mice and WT littermates revealed no significant differences in body weight, and fat and lean mass composition (Figure 1). Even though a trend toward a greater body lean mass was observed in VPAC1^−/−^ mice, those values were not significant at the end of the 12-week study (Figure 1C). In a previous study, VPAC1^−/−^ mice were observed to have developmental delays with lower body weight, similarly to VIP null mice [28], with significantly lower body weights observed in 4-week-old VPAC1^−/−^ male and female pups, compared to WT and heterozygous mice. In our laboratory, we have also observed lower body weights and higher mortality in VPAC1^−/−^ pups (unpublished data), similarly to Fabricius et al. [8]. At the beginning of our study, VPAC1^−/−^ mice at 8 weeks of age had body weight averages 3.4 g lower than WT littermates, yet not statistically significant, but after 12 weeks, VPAC1^−/−^ and WT body weights were similar.

Previously, we demonstrated that VIP^−/−^ mice had a leaner body phenotype with deficit of fat mass accumulation as they aged, and a better maintained body lean mass [16]. Other authors have shown that VPAC2^−/−^ male mice had lower body weight and length at 8 weeks of age, but they accumulated fat mass as they aged [33]. This leaner body phenotype described in VPAC2^−/−^ mice is similar to the one that we described in VIP^−/−^ mice [16]. However, in the current study we did not observe a leaner phenotype in VPAC1^−/−^ mice, thus suggesting that VPAC1R is not responsible for the phenotypic changes observed in VIP^−/−^ mice [16]. Other authors reported [34] that WT mice treated with a VPAC1 agonist were protected against high-fat-diet-induced obesity. Furthermore, it was published that a treatment with VPAC1 blocking antibodies for 15 weeks in WT mice had no significant impact on adipogenesis and weight gain [35]; therefore, these in vivo studies did not reach similar conclusions. An in vitro study by Akesson et al. [36] reported in rat adipocytes that PACAP, VIP, as well as two different VPAC2-R agonists, induced lipolytic effects, thus indicating that even though VPAC1R, VPAC2R, and PAC1R were all expressed on adipocytes, only VPAC2R mediated VIP- and PACAP-induced lipolysis. These data are concordant with our in vivo findings that body fat mass was not affected by genetic lack of VPAC1R in mice. According to Lijnen et al., inhibition of VPAC1 receptors by monoclonal antibodies in WT mice, fed a high-fat diet to induce obesity for 15 weeks, failed to modify weight gain and fat mass, but induced adipocyte hypertrophy in the subcutaneous compartment [35]. These data are consistent with our current data on VPAC1^−/−^.

In our study, after 12 weeks of phenotype development observation, a complete feeding and metabolic profile analysis was conducted by indirect calorimetry in VPAC1^−/−^ and WT mice. Our results revealed no significant differences in food consumption and amount of time spent eating in the two experimental groups; however, a significantly increased number of feeding bouts was found in VPAC1^−/−^ mice during the active phase (dark cycle) (Figure 2A). Other researchers have shown that a long-term treatment with a VPAC1 agonist inhibited food intake significantly over a 28-day experimental period [34]. On the other end, Alexander at al. reported in a rat study that a pretreatment with a VIP antagonist did not affect food intake [37], therefore, these data would concur with our feeding data in VPAC1^−/−^ mice, as well as with our previously published data in VIP^−/−^ mice, showing no difference in food intake from WT littermates 16]. Bechtold et al. [38] found in a VPAC2^−/−^ murine model no significant differences in food consumption, except for a significant decrease in hopper activity during the dark cycle compared to WT; whereas, other authors reported a lower daily food intake in VPAC2^−/−^ mice [38]. The same studies [38,39] demonstrated an altered physiology in VIP^−/−^ and VPAC2^−/−^ mice, with 3–4 h advancement in their metabolic and feeding rhythms, due to an altered expression of peripheral clock genes at feeding time in the VPAC2^−/−^ mice liver. Our current data confirm that VPAC1Rs do not affect clock genes and circadian rhythm of feeding.

Our analysis demonstrated an increased number of feeding bouts in VPAC1^−/−^ mice, which could be explained by VPAC1R localization on the taste receptive cells of the tongue and by the altered taste cells and preferences in VIP^−/−^ mice [40]. These data would suggest that lack of VPAC1R expression could potentially change the food preference/aversion behavior without impacting satiety/hunger and food consumption in mice.

An indirect calorimetry analysis conducted in VPAC1^−/−^ mice revealed lower total energy expenditure (TEE) during both the active (dark) and dormant (light) cycle (Figure 3A,B). No significant differences in physical activity parameters, including total movement, fine activity (scratching/sniffing), and wheel activity (Figure 4) were detected.

Our study is the first to evaluate VPAC1R impact on TEE and physical activity levels, and the results would suggest that VPAC1^−/−^ mice can maintain normal levels of physical activity while requiring less metabolic energy. Even though VIP is a potent vasodilator, the administration of VIP, or a VIP agonist, has been shown to dilate coronary vasculature without impacting the mouse heart rate or myocardial contractility [41,42], thus, these data would support the normal physical activity parameters measured in our study.

A full metabolic profile assessment of VPAC1^−/−^ and WT mice at 12 weeks of age revealed similar respiratory quotient (RQ) values (Figure 3C,D), but significant differences in oxygen consumption (Figure 3E,F), carbon dioxide production (Figure 3G,H), and water vapor production (Figure 3I,J) between the two groups of mice. In fact, VPAC1^−/−^ mice had lower VO_2_ and VCO_2_ during both the dark (active) and light (dormant) phase, and lower VH_2_O only during the dark cycle. This is the first study that addresses the effects of VPAC1R on respirometry. In the literature, VIP administration in a perfused rat heart did not impact VO_2_ [42]. In our study, VPAC1^−/−^ mice had significantly decreased VO_2_. Other authors reported lower VO_2_ and VCO_2_ values in VPAC2^−/−^ mice during the active phase (dark cycle) without significant changes in RQ during both the active and dormant phase [38]. These findings are complementary to ours and suggest that both VPAC1 and VPAC2 receptors are involved in the regulation of respiratory gases and in the maintenance of metabolic homeostasis.

Circadian rhythm alterations have been documented in mice lacking certain clock genes, and VIP has been shown to mediate the synchronization of these rhythms [43,44,45]. Clock genes regulate metabolism by modulating the timing of food consumption and feeding rhythms [44,46]. Light has been shown to be a key regulatory factor in VIP/VPAC2 signaling pathways, thus establishing that this ligand–receptor pair alters the clock function [44]. VPAC2R is implicated in the regulation of these homeostatic rhythms centrally, through binding of VIP in the suprachiasmatic nucleus [44,47,48,49]; however, limited research has been conducted on the role of VPAC1R in modulating circadian rhythm signals. Centrally, VPAC2R and PAC1R mRNA are expressed in the rat suprachiasmatic nucleus (the regulatory center of the circadian clock), whereas VPAC1R mRNA expression is absent [49]. In the current study, the absence of alterations in circadian signaling confirms that VPAC1R are not involved in these mechanisms.

In the current study, plasma levels of a panel of orexigenic and anorexigenic metabolic hormones were measured in VPAC1^−/−^ and WT mice, during both fasting and postprandial conditions (Figure 5) to assess potential endocrine alterations in peripheral signals controlling the balance between orexigenic and anorexigenic responses and glucose homeostasis. Plasma levels of active-Ghrelin were not significantly different in VPAC1^−/−^ mice, even though higher postprandial ghrelin values, nearing significance, were measured in VPAC1^−/−^. Centrally, food intake and appetite are modulated by hormone signaling and binding within the hypothalamic ARC nucleus [49]. Ghrelin is a key hunger hormone, released peripherally to stimulate food intake and fat storage in times of depleted plasma blood glucose [50,51]. In VPAC2^−/−^ mice, circadian alterations, changes in anticipatory rhythms of activity and hormone secretion were described [38].

Here, VPAC1^−/−^ fasting and postprandial active-Ghrelin levels were not significantly different; thus, these data are concordant with the food consumption and would suggest that VPAC1R is not directly involved in the hunger initiation mechanism, even though VIP has been implicated in these pathways, as shown by the altered hormones that we reported in VIP^−/−^ mice [16].

VPAC1^−/−^ mice had significantly higher plasma levels of GLP-1 during both fasting and postprandial conditions. In the literature, there are no other reports of GLP-1 plasma values in a VPAC1 null model. However, we have previously shown higher fasting and postprandial levels of GLP-1 in VIP^−/−^ mice [16]. Therefore, these data suggest that VPAC1R could mediate VIP inhibitory effects on GLP-1 secretion. GLP-1 plays an important role in glucose homeostasis by increasing insulin and suppressing glucagon secretion in response to blood glucose changes [52,53]. In the present study, no significant differences in plasma glucagon levels were found in VPAC1^−/−^ during fasting; however, glucagon was significantly higher during postprandial conditions (Figure 5C). We have previously [16] described significantly higher postprandial level of glucagon in VIP^−/−^ mice, therefore, this would suggest that VIP could inhibit the postprandial release of glucagon through VPAC1R. Glucagon stimulates glucose blood increase by promoting glycogenolysis and gluconeogenesis, working together with insulin to maintain glucose homeostasis [54]. In VPAC1^−/−^ mice, no significant differences in insulin levels were measured during either fasting or post-prandial conditions (Figure 5D), thus, these data support our previous findings in VIP^−/−^ mice [16]. VPAC1R has been indicated as a key player in protecting mice against diabetes-associated inflammation and oxidative stress [55]. VPAC1R mRNA has higher expression than VPAC2R and PAC1R in murine diabetic pancreas [55,56]. In diabetic mice, a 28-day treatment with a VPAC1 agonist decreased plasma glucose levels and reduced oxidative stress [56]. Another study reported that a VPAC1R agonist increased hepatic glucose production, whereas a VPAC2 agonist elevated plasma insulin levels [57]. De Vadder et al. reported that VIP activated intestinal gluconeogenesis [54], and Erendor at al. [58], using VIP encoded by a lentiviral gene therapy vector, suppressed diabetes-related inflammation and increased beta-cell proliferation. In our VPAC1R^−/−^ mice, postprandial glucagon levels were higher, suggesting that VPAC1R could play a key role in GLP-1 and glucagon release, thus contributing to plasma glucose homeostasis.

We reported in VPAC1^−/−^ mice significantly lower postprandial, but not fasting, leptin levels. Anorexigenic hormones, such as leptin, regulate intake, digestion, and absorption of nutrients by binding to gut receptors to maintain caloric homeostasis [59,60].

Previously, we reported in VIP^−/−^ mice significantly higher fasting leptin levels, a disrupted feeding pattern and altered food consumption [16]. Asnicar et al. also found higher levels of leptin in VIP^−/−^ mice [33]. Therefore, here, we propose that the VPAC1R is involved in the physiologic postprandial increase of leptin, but not in the inhibition of leptin secretion during fasting. No studies have analyzed the role of VPAC2 on leptin plasma levels.

In our current research, PYY plasma levels were significantly higher in VPAC1^−/−^ mice during both fasting and postprandial conditions.

PYY, like leptin, is a peripheral anorexigenic hormone, released by gastrointestinal L cells [61,62]. These data are in concordance with the higher levels of PYY that we reported in VIP^−/−^ mice during both fasting and postprandial conditions [16]. VIP is known to mediate the colonic release of PYY [63], therefore, these results support a VPAC1R-mediated inhibitory mechanism in PYY fasting and postprandial plasma release.

To further assess glucose homeostasis in VPAC1^−/−^ mice, we performed glucose and insulin tolerance tests. VPAC1^−/−^ mice showed no significant differences in blood glucose levels following IP injection of glucose, however, after insulin IP administration, they had significantly lower blood glucose at 60 and 120 min (Figure 6). Our data are in concordance with a study in which hypoglycemia was reported after insulin injection in VPAC1 null mice [28]. Therefore, our data suggest that VPAC1R is involved in the regulation of blood glucose homeostasis. In Fabricius at al. [28], a significant hypoglycemia was observed also in fasted VPAC1^−/−^ mice, orally administered with glucose. The observed differences might be explained by the different route of administration, oral vs. intraperitoneal. Since VPAC1, VPAC2, and PAC1 receptors are all expressed on pancreatic islet cells, where they bind to VIP and PACAP [55,64], further research is necessary to clarify the mechanisms by which these receptors modulated insulin secretion.

## 5. Conclusions

The strength of the current study is that this is the first complete characterization of the phenotypic, metabolic, calorimetric, and homeostatic effects of VPAC1R in a null murine model. Lack of VPAC1R gene expression in mice significantly lowered total energy expenditure, respiratory gases, and altered glucose/insulin regulation and plasma metabolic hormone levels. Our findings support a key role for VPAC1R in the regulation of metabolism, energy, and glucose homeostasis through the control of plasma GLP-1, glucagon, leptin, and PYY levels. A weakness of the current study is its reliance on an animal model, however the results support the need for further studies to explore the effects of VPAC1R in models of obesity, diabetes mellitus, and metabolic syndrome by using VPAC1 null genetic models, or VPAC1 agonists and antagonists in WT animals, in order to develop future potential pharmacological protocols.

## Data Availability

Not applicable.

## Abbreviations

CNS	central nervous system
GI	gastrointestinal
GLP-1	glucagon-like peptide 1
IGF	insulin-like growth factor
PAC_1_-R	pituitary adenylate cyclase activating polypeptide type 1 receptor
PACAP	pituitary adenylate cyclase activating polypeptide
PCR	polymerase chain reaction
PYY	peptide YY
SEM	standard error of the mean
VIP	vasoactive intestinal polypeptide
VPAC1R	vasoactive intestinal polypeptide receptor 1
VPAC2R	vasoactive intestinal polypeptide receptor 2
BW	body weight
qPCR	quantitative polymerase chain reaction
T2D	type 2 diabetes
TEE	Total Energy Expenditure
VCO_2_	carbon dioxide production
VO_2_	rate of oxygen consumption
VH_2_O	water vapor
RQ	respiratory quotient
GPCRs	G-protein coupled receptors
WT	wild type
GTT	glucose tolerance test
ITT	insulin tolerance test
